# Medicaid managed care and preventable emergency department visits in the United States

**DOI:** 10.1371/journal.pone.0240603

**Published:** 2020-10-29

**Authors:** Mohammad Usama Toseef, Gail A. Jensen, Wassim Tarraf

**Affiliations:** 1 Institute of Gerontology, Wayne State University, Detroit, MI, United States of America; 2 Department of Economics, Wayne State University, Faculty/Administration Bldg, Detroit, MI, United States of America; 3 Department of Healthcare Sciences, Wayne State University, Detroit, MI, United States of America; Charite Universitatsmedizin Berlin, GERMANY

## Abstract

**Objectives:**

In the United States the percentage of Medicaid enrollees in some form of Medicaid managed care has increased more than seven-fold since 1990, e.g., up from 11% in 1991 to 82% in 2017. Yet little is known about whether and how this major change in Medicaid insurance affects how recipients use hospital emergency rooms. This study compares the performance of Medicaid health maintenance organizations (HMOs) and fee-for-service (FFS) Medicaid regarding the occurrence of potentially preventable emergency department (ED) use.

**Methods:**

Using data from the 2003–2015 Medical Expenditure Panel Survey (MEPS), a nationally representative survey of the non-institutionalized US population, we estimated multivariable logistic regression models to examine the relationship between Medicaid HMO status and potentially preventable ED use. To accommodate the composition of the Medicaid population, we conducted separate repeated cross-sectional analyses for recipients insured through both Medicaid and Medicare (dual eligibles) and for those insured through Medicaid only (non-duals). We explicitly addressed the possibility of selection bias into HMOs in our models using propensity score weighting.

**Results:**

We found that the type of Medicaid held by a recipient, i.e., whether an HMO or FFS coverage, was unrelated to the probability that an ED visit was potentially preventable. This finding emerged both among dual eligibles and among non-duals, and it occurred irrespective of the adopted analytical strategy.

**Conclusions:**

Within the U.S. Medicaid program, Medicaid HMO and FFS enrollees are indistinguishable in terms of the occurrence of potentially preventable ED use. Policymakers should consider this finding when evaluating the pros and cons of adopting Medicaid managed care.

## Introduction and background

In the United States (U.S.), Medicaid is a public health insurance program that covers roughly 70 million low-income and financially needy Americans. Since the early 1990s state governments, which administer Medicaid, have increasingly turned to “managed care” as a way to structure Medicaid insurance and provide for the delivery of health services. Managed care also appears in other publicly funded health insurance programs in the US like Medicare Advantage and private delivery of subsidized coverage through the marketplace. Nationwide, the percentage of Medicaid recipients enrolled in some form of managed care has increased more than seven-fold since 1990, up from 11% in 1991 to 82% in 2017 [1, 2]. Yet little is known about whether and how this major change in the structure of Medicaid affects recipients’ use of hospital emergency departments (EDs). This paper examines this issue.

Managed care within Medicaid encompasses a broad array of contract arrangements between state Medicaid programs and private companies. All forms of Medicaid managed care share in common insurance rules for accessing care and the actual provision of health services are *more integrated* than under conventional fee-for-service (FFS) Medicaid [3]. The most prevalent forms of Medicaid managed care are Medicaid health maintenance organizations (HMOs); the other two forms of Medicaid managed care are primary care case management (PCCM) and limited-benefits plans [4]. Medicaid HMOs, typically, are private-sector HMOs that contract with Medicaid to provide the delivery of health services to Medicaid recipients in exchange for a fixed fee per recipient per month (capitation). These HMOs link each Medicaid enrollee with a primary care physician (PCP) who serves as a care coordinator and gatekeeper for the enrollee’s access to services. Because a Medicaid HMO is paid via capitation, it should have incentives to structure its payments to providers and its rules for PCPs and enrollees so that health services are delivered cost-effectively with PCP oversight and continuity of care.

States pay HMOs’ capitation fees rather than act themselves as health insurers for Medicaid. States have turned to managed care for several reasons, including: (1) achieving greater control and predictability over their spending on Medicaid, (2) improving accountability for program performance, access to services, and care quality, and (3) improving care coordination, reducing duplicate and/or unnecessary services, and promoting greater efficiency in care provision [3]. Unlike FFS Medicaid, paying for care through capitation might also deter physicians and hospitals from possibly oversupplying services of questionable benefit as a way of enhancing revenue [5].

Several countries with national healthcare systems have also introduced various forms of managed care in their programs, including the United Kingdom, the Netherlands, Germany, Switzerland, Israel, Estonia, Latvia, and Lithuania [5–9]. Outside the U.S., however, managed care models are often described using different terminology, including “integrated care,” “shared care,” “transmural care,” and “comprehensive care” [10].

Ideally, managed care plans aim to reduce costs, provide preventive services and discourage the overutilization of healthcare resources via care provided by the PCP or the gatekeeper. Thus, it is reasonable to expect that Medicaid recipients in managed care plans might have fewer ED visits, and have a lower prevalence of ED visits which might have been “potentially preventable,” compared to recipients with conventional Medicaid. Estimates suggest that around 13% to 27% of ED visits in the United States could instead have been managed and taken care of in physician offices, clinics and urgent care centers [11]. These potentially preventable ED visits are associated with ambulatory care sensitive conditions (ACSCs) and are considered to be an indicator of health care quality [12, 13]. Although ED use for ACSCs is not the only measure of assessing health care quality, it is an important measure of quality particularly when preventable. As such, it is important to assess whether managed care within Medicaid, given its gatekeeper structure, is capable of better controlling preventable ED utilization as compared to conventional Medicaid.

In this study, we use nationally representative data on Medicaid recipients to examine the association between type of Medicaid coverage and recipient visits to the ED. We address the following question: Do Medicaid recipients in managed care plans use ED services differently than recipients in conventional fee-for-service (FFS) Medicaid, and if so, in what ways? Comparing FFS and HMO recipients within Medicaid is complicated by the fact that there are two distinct subpopulations that tend to differ dramatically in terms of their demographics and medical needs. The first, includes recipients whose only source of health insurance is Medicaid, the other, encompasses recipients who are eligible for both Medicare and Medicaid (called “dual eligibles” or “duals”). Compared to recipients with Medicaid only (called “non-duals”), duals typically have greater and more complex healthcare needs, including more mental and physical disabilities and multiple chronic conditions, and although duals comprise just 15% of all Medicaid enrollees, they account for 32% of all Medicaid spending [14–16]. Because of their very different health needs, these two groups may use the hospital ED differently. To address this issue, we conduct separate analyses of the effects of Medicaid HMOs for each group, i.e., one analysis for duals, another analysis for non-duals.

There is some limited evidence that managed care plans within Medicaid, especially Health Maintenance Organizations (HMOs), are associated with a decrease in ED utilization [17–22]. However, two more recent studies found that Medicaid managed care market penetration is associated with an increase in ER use [23, 24]. A recent study by Hu and colleagues assessed the impact of mandatory Medicaid managed care implementation in Florida on preventable ED visits but its focus was on racial/ethnic disparities in such visits [25]. They found that making managed care mandatory for Medicaid recipients slowed the growth of preventable ED visits for minorities relative to whites. No studies, to our knowledge, have used nationally representative data to examine whether and how a recipient’s type of Medicaid coverage, e.g., whether managed care or conventional Medicaid, affects their preventable ED utilization. This study does so, and examines the occurrence of potentially preventable ED visits.

Our paper contributes to the literature in three ways. First, instead of focusing on a single or a few states, we analyze nationally representative data from the Medical Expenditure Panel Survey (MEPS) spanning 2003–2015 on all nonelderly U.S. adults with Medicaid. Over this period, the percentage of Medicaid recipients under some form of managed care grew from around 58% to 75% [26, 27]. Second, since there is potential for managed care to enroll healthier patients and discourage participation of high utilizers, we explicitly address the possibility of selection bias into Medicaid HMOs by using propensity score weighting. Third, and most importantly, we pay close attention to potential differences by dual-eligibility status, and as such we stratify our analyses to examine whether the effects of Medicaid managed care plans differ between dual eligibles and non-duals. Whether there are differential effects of Medicaid managed care on ED use based on dual vs. non-dual status has not been studied before. Attending to potential differences is important given the greater health needs and higher healthcare spending among dual eligibles.

## Methods

### Study design

We used data from the Household Component (HC) of the Medical Expenditure Panel Survey (MEPS), a nationally representative survey of the non-institutionalized U.S. population. The MEPS, conducted by the U.S. Agency for Healthcare Research and Quality (AHRQ), is a set of large-scale, ongoing, surveys which collect data from families and individuals regarding their use of health services. By merging together the HC full-year consolidated data files and the HC emergency room visits files, we gathered information on all the MEPS participants who used the ED.

This study analyzed thirteen years of the MEPS data covering 2003–2015. We focused on the MEPS respondents with Medicaid coverage, ages 18 to 64, and further divided this sample into two subgroups: non-duals who had only Medicaid throughout the year of their interview, and duals who had both Medicare and Medicaid throughout that year. All MEPS data are publicly available and obtainable from the MEPS AHRQ website: https://www.meps.ahrq.gov/mepsweb/data_stats/download_data_files.jsp. This study uses secondary analyses of publicly available de-identified data and as such is exempt from Institutional Review Board approval.

### Measures

#### Outcome variables

Our outcome of interest was whether a respondent had at least one ED visit in a year that was potentially preventable or not (1 if yes, 0 otherwise). Over the period of our sample, medical conditions associated with a respondent’s ED visits were documented by the MEPS interviewers as verbatim text and then coded by professional coders into International Classification of Diseases, Ninth Revision (henceforth referred to as ICD-9 codes). These ICD-9 codes were further aggregated into Clinical Classification Categories (CCCs) [28]. According to AHRQ, these codes were verified and error rates for each coder did not exceed 2.5 percent [28, 29]. Additionally, there could be multiple codes for each visit and these were not reported in order of importance or severity. The MEPS stopped reporting the ICD-9 codes after 2012, however it continued to report the CCCs till 2015; since the MEPS have adopted the ICD-10 coding system from 2016, ICD-10 mapping to CCCs is still under review [30].

An ED visit was determined to be potentially preventable using guidelines provided by AHRQ [13]. The Prevention Quality Indicators (PQIs), developed by the AHRQ, identify quality of care for ACSCs using hospital discharge data. If any code of a particular ED visit corresponded to an ACSC, then that ED visit was determined to be potentially preventable. In all, 12 adult ACSCs were used to flag potentially preventable ED visits: bacterial pneumonia, dehydration, urinary tract infection, perforated appendix, angina without procedure, congestive heart failure, hypertension, asthma, chronic obstructive pulmonary disease, uncontrolled diabetes, diabetes complications and amputations among patients with diabetes. As mentioned earlier, ED visits for these conditions could have been prevented with adequate primary care.

Once respondents with missing values for covariates were excluded from the sample, the sample size for our analysis of potentially preventable ED visits included 6,877 non-duals and 1,133 duals.

#### Primary predictor

The primary predictor is a binary indicator of whether the recipient was enrolled in a Medicaid HMO (1 if yes, 0 otherwise).

#### Other variables

In addition to Medicaid HMO enrollment, all estimated models controlled for a rich set of additional covariates, including the recipient’s demographic characteristics and socioeconomic status (SES), their use of preventive health care services, health and functional status, presence of various health conditions, and their attitudes towards health insurance and risk-taking. Demographic and SES indicators include age (18–35, 36–55 and 56–64), region (Northeast, Midwest, South and West), gender, education (high school or less, some college and college or more) and income level (poor, near poor, low income and middle or high income). Health and functional status variables included self-reported health and self-reported mental health (excellent, very good, good and fair or poor), received help or supervision for activities of daily life (both instrumental and general), their body mass index (BMI), current smoking status (yes or no), and whether or not they have access to a usual source of care. Health conditions controlled for in all models included diabetes, asthma, high blood pressure, coronary heart disease, angina, myocardial infarction, stroke, any other heart condition and emphysema. Due to their high prevalence, AHRQ refers to these conditions as priority conditions [31].

To gauge how conscientious an individual was about taking care of their own health, five facets of preventive care were also included in the models, including whether, within the last year, they had a cholesterol check, a flu shot, a routine check-up, and whether they had been advised by a doctor to restrict their intake of fatty food, or advised by a doctor to exercise more.

A recipient’s attitude towards insurance and risk-taking are measured using four variables which gauge whether they agree with the following four statements (considered one at a time): “I’m healthy enough that I really don’t need health insurance,” “Health insurance is not worth the money it costs,” “I’m more likely to take risks than the average person,” and “I can overcome illness without help from a medically trained person.”

### Data analysis

All analyses accounted for the complex survey design and survey weights provided by the MEPS using the survey specific commands and functions in Stata v.15 software [32].

Analysis proceeded in four steps. First, we generated descriptive statistics to examine and test for differences in sample characteristics based on recipients’ dual-eligibility status. Survey design based t-tests and chi-square tests were used to determine whether differences exist in ED use and other covariates between duals and non-duals. Upon finding strong evidence that duals and non-duals are very different populations, we chose to conduct separate analyses for each subgroup. In the second step, we generated descriptive statistics based on Medicaid HMO enrollment status. We did this to examine differences between Medicaid HMO and FFS enrollees. Third, we estimated separate multivariable logistic regressions for duals and non-duals to examine the relationship between preventable ED visits and Medicaid managed care status, controlling for all the covariates described earlier. Finally, we re-estimated all models, applying Inverse Probability of Treatment Weighting (IPTW) using propensity scores to control for possible selection bias in HMO plan enrollment [33]. The presence of favorable self-selection into Medicaid managed care plans has been documented [1, 34, 35], and if not addressed, could lead to bias in the estimated effects of Medicaid managed care [36, 37]. We followed Austin (2011) and Dugoff et al. (2014) to estimate propensity scores, to generate weights and to combine those weights with the MEPS survey weights to form a set of new weights for individuals in our sample [33, 38]. More specifically, we regressed Medicaid HMO enrollment on all other covariates (using logistic regression) and derived the predicted probability from the fitted regression model; this predicted probability is the propensity score, *e*_*i*_ for individual *i*. Using *e*_*i*_ we generated weights for each individual, *w*_*i*_: (*Medicaid HMO*)/*e*_*i*_ + (*Medicaid FFS*)/*(1-e*_*i*_). To consider the MEPS survey weights, we multiplied *w*_*i*_ with the MEPS survey weight [38]. Details regarding propensity score estimation and IPTW techniques can be found elsewhere [39–45].

### Sensitivity analysis

As noted earlier, the MEPS did not report ICD-9 codes after 2012, and so we used the CCCs to identify preventable ED visits. This however introduces imprecision in our analysis as a CCC may include ICD-9 codes that are not associated with preventable ED visits. Therefore, as a sensitivity analysis, we conducted the same analysis with two outcome variables of preventable ED visits based on ICD-9 codes and CCCs separately. This restricted our time frame to the year 2012. We also re-estimated the original models by adding year fixed effects to help account for regional or nationwide shifts in Medicaid managed care enrollee composition over time.

## Results

### Differences between duals and non-duals

Compared to non-duals, duals were more likely to have preventable ED visits during the year (25.3% vs 17.9%; p < 0.0001), and were less likely to be enrolled in a Medicaid HMO (31.2% vs. 49.7%; p < 0.0001). Duals and non-duals also differed on all of the covariates in our analysis, with the exception of income and smoking status. Specifically, duals were more likely to be older, to report worse levels of both physical and mental health, to be receiving help with activities of daily living, and to report having chronic medical conditions. Duals were also more likely to use preventive health care services. These differences, displayed in S1 Table, warrant analyzing duals and non-duals separately.

### Differences between HMO and FFS recipients

Among non-duals, we found some important differences in characteristics of HMO and FFS Medicaid recipients. As shown in Table 1, we found statistically significant differences in region, income, self-reported physical health, BMI, access to usual source of care, almost all the preventive care services utilization, attitudes towards insurance and risk variables, and some clinical conditions (asthma and high blood pressure). Among duals the characteristics of HMO and FFS Medicaid recipients were statistically different for use of cholesterol check only.

**Table 1:** Characteristics of the medicaid population with preventable Emergency Department (ED) admission ages 18–64 by dual-eligibility^a^ and Medicaid HMO^b^ status. Results are based on aggregated data from the Medical Expenditures Panel Survey^c^.

	Preventable ED					
	Non-duals			Duals		
Characteristics (%)	FFS	HMO	p-value	FFS	HMO	p-value
Preventable ED Admission	18.2	17.2	0.4025	24.4	27.9	0.3208
**Demographics**						
**Age**			0.9467			0.9398
18 to 35	55.9	56.4		11.2	10.7	
36 to 55	35.2	34.9		57.8	59.3	
56 and above	8.9	8.8		31	29.9	
**Region**			<0.001			0.0968
Northeast	18.7	26.5		18.4	22.4	
Midwest	26.1	23.6		25	16.4	
South	34.2	24.9		39.1	37.9	
West	20.9	25.1		17.5	23.3	
**Male**	28.6	25.6	0.0791	41.7	34.9	0.107
**Education**			0.7961			0.3168
High School or Less	31.9	32.5		25.7	29.7	
Some College	46.7	47		47.6	41.3	
College or More	12.4	20.5		26.8	29	
**Income**			<0.001			0.1999
Poor	50.3	56.7		48.7	56.8	
Near poor	9.1	10.1		12.8	12.2	
Low Income	20.3	18.3		20.3	17.7	
Middle or High Income	20.3	15		18.2	13.3	
**Health and Functional Status**						
**Self-Reported Physical Health**			0.0145			0.1822
Excellent	15.1	11.9		2.6	4.8	
Very Good	21.9	21.3		10.6	7.3	
Good	28.4	30.2		21.4	21.7	
Fair/Poor	34.6	36.6		65.4	66.2	
**Self-Reported Mental Health**			0.6973			0.0933
Excellent	27.4	26.7		10.4	9	
Very Good	20.6	21.7		18.8	13.2	
Good	29.6	30.4		29.2	36.3	
Fair/Poor	22.5	21.2		41.7	41.5	
**IADL**^f^	8.9	7.6	0.1707	27.9	26.2	0.6237
**ADL**^g^	4.7	3.6	0.0893	12.9	14.1	0.6651
**BMI**			0.0169			0.6089
Underweight	3.2	2.4		1.3	1.5	
Normal	30.8	26.4		20	23.8	
Overweight	25.5	26.9		29.2	24.9	
Obese	40.5	44.3		49.5	49.7	
**Currently Smoke**	40.7	39.9	0.652	43.1	50.4	0.1006
**USC**^h^	73.9	79.8	0.00012	92.5	93.1	0.3347
**Preventive Care Services Utilization**					
Cholesterol Check	46.7	41.3	0.0058	19.3	12.7	0.012
Flu Shot	69.2	67.7	0.3401	44.2	46.8	0.5262
Routine Check	35.1	27.5	<0.001	15	14.44	0.8413
Restrict Fatty Food	30.7	37	<0.001	53.5	52.7	0.8431
Exercise More	41.2	46.3	0.002	57.2	59.2	0.6018
**Attitudes towards health insurance and risk**						
**Agree With Following Statements**						
Do not need health insurance	16.6	13.3	0.0045	8	6.9	0.5888
Insurance not worth cost	43.3	38.6	0.0059	34.6	30.8	0.3557
More likely to take risks	41.1	39.6	0.3485	38.4	38.3	0.9732
Overcome illness with no help	31.1	27.4	0.0123	18	18.3	0.9225
**Clinical Conditions**						
Diabetes	11.5	12.7	0.2631	29.3	29.9	0.8976
Asthma	20.7	22.5	0.2287	28.9	30.9	0.5617
High Blood Pressure	31.9	32.8	0.5498	65	61.5	0.4076
Coronary Heart Disease	5.1	5.5	0.5739	14.1	14.6	0.8673
Angina	3.3	3.6	0.6255	9.1	10.4	0.6248
Myocardial Infarction	4.7	5	0.7389	12.1	12.5	0.8888
Any other heart disease/condition	11.5	10.8	0.5204	27.4	23.3	0.313
Stroke	5.4	5.1	0.7398	13.2	14.2	0.6766
Emphysema	4.5	2.8	0.0129	13.2	12.7	0.8795

a. Dual Eligibility Status: Non-Duals are Medicaid recipients whose only health insurance is Medicaid. Duals are Medicaid

recipients who are also insured through Medicare.

b. HMO = Health Maintenance Organization

c. Data source: Public use data files from the Medical Expenditure Panel Survey (MEPS) for 2003 through 2015.

d. FFS = Fee For Service

e. p-values from Survey Design Based F-test

f. Received help or supervision for instrumental activities of daily living

g. Received help or supervision for activities of daily living

h. Usual source of care

i. Attitudes towards health insurance and risk

### Medicaid HMO Status and the odds of preventable ED visits

Table 2 (and S2 and S3 Tables) presents our findings regarding the association of Medicaid HMO status and the odds of having preventable ED visits during the year controlling for other covariates. Regardless of our method of estimation, among non-duals we found no relationship between the type of Medicaid held by the recipient, i.e., whether an HMO or conventional FFS, and the occurrence of preventable ED visits during the year, after controlling for other possible determinants of such visits. The same finding emerged in the sample of duals, i.e., the models estimated suggest there is no relationship between the type of Medicaid plan held by the recipient and whether he or she had preventable ED visits. However, among both non-duals and duals, there were some significant predictors of having preventable ED use. For non-duals, males had significantly lower odds of having preventable ED visits, while worse physical health status was associated with significantly higher odds of having preventable ED visits. For duals, individuals who were advised by a doctor to restrict fatty foods had significantly higher odds of having preventable ED visits.

**Table 2:** Association between medicaid Health Maintenance Organizations (HMO) coverage and Emergency Department (ED) visits among non-duals and dual eligibles^a^, ages 18–64. Results are based on data from the Medical Expenditures Panel Survey^b^.

	Among Non-Duals	Among Duals
**Preventable ED Visits**		
**Survey-Weighted Logistic Model**		
Odds Ratio^c^	0.911	1.226
(95% CI^d^)	(0.778–1.068)	(0.851–1.767)
**Propensity Score Weighted Model**		
Odds Ratio^e^	0.917	1.209
(95% CI^d^)	(0.780–1.078)	(0.846–1.728)

Notes:

a. Dual Eligibility Status: Non-Duals are Medicaid recipients whose only health insurance is Medicaid. Duals are Medicaid recipients who are also insured through Medicare.

b. Data source: Public use data files from the Medical Expenditure Panel Survey (MEPS) for 2003 through 2015.

c. Adjusted odds ratios from a multivariable logit regression estimated with survey weights, which controls for Medicaid HMO enrollment, demographics, health and functioning, attitudes towards health insurance and risk, preventive care services utilization, and clinical conditions.

d. CI = Confidence Interval

e. Adjusted odds ratios from a multivariable logit regression estimated with propensity score weights, which controls for Medicaid HMO enrollment, demographics, health and functioning, attitudes towards health insurance and risk, preventive care services utilization, and clinical conditions.

### Sensitivity

In the sensitivity analyses, we restricted our time frame to 2012 so we could construct separate preventable ED visit variables based on ICD-9 codes and CCCs. The sensitivity analyses yielded similar results as the main analyses and thus we found no significant association between Medicaid HMO enrollment and preventable ED visits. Results from the sensitivity analysis are presented in S4–S7 Tables. Therefore, even when we used the more precise ICD-9 codes and different estimation methods, the results did not change. Furthermore, our results did not change when we included year fixed effects in our original models (results available from authors on request). This suggests that our results were robust to changes in different model specifications and outcome variable definitions.

## Discussion

From this nationally representative sample of Medicaid recipients, ages 18–64, in the U.S. we found that the type of Medicaid held by a recipient, i.e., whether an HMO or FFS coverage, was unrelated to the probability that an ED visit was potentially preventable. This finding emerged among dual eligibles and among non-duals, and it occurred irrespective of the adopted analytical strategy. Dual eligibles were more likely to have potentially preventable ED visits than non-duals, however, within each of these Medicaid subpopulations, there were no differences in the odds of experiencing potentially preventable ED use based on Medicaid HMO enrollment. This suggests that Medicaid HMO and FFS enrollees are indistinguishable in terms of their potentially preventable use of EDs.

Previous studies have found mixed evidence regarding the effect of Medicaid managed care on overall ED use [17–24]. This is somewhat surprising given that HMO enrollees (among both duals and non-duals) more often report having a usual source of care (see Table 1), and other studies have shown that, at least in more general populations (not specific to Medicaid recipients), having a usual source of care is associated with having fewer ED visits, including non-emergent visits [46, 47]. One possible explanation for why we did not find any effects for HMOs is that all of our models also controlled for whether the recipient reported having a usual source of care, and that variable might have picked up some of the effects of being in an HMO. To assess whether this is what happened we re-estimated all of our models, this time excluding the variable that measures having a usual source of care. Our findings remained unchanged, i.e., being in a Medicaid HMO still had no effects on potentially preventable ED utilization. Another possibility is that we failed to find effects of HMOs because we ran separate analyses for duals and non-duals, rather than pooling them together, as most previous studies have done. (Recall, duals have higher rates of ED use but are less likely to have Medicaid HMO coverage). To assess the validity of this explanation, we re-estimated all of our models, this time pooling duals and non-duals together. For these models too our findings remained unchanged.

In the case of ED visits for ACSCs, we found no differences between Medicaid HMO and FFS Medicaid recipients. Previous work suggests that such visits may not necessarily be a result of poor judgement on the part of patients, but instead may indicate that they have poor access to quality primary patient centered and continuous care [48, 49]. For example, it is not uncommon for office staff in primary care practices and clinics to advise Medicaid patients who call in seeking treatment for a minor problem to go to the ED instead [49]. This may be happening because there is simply more demand for primary care services than the U.S. healthcare system can handle due to the continuing shortage of PCPs and PCP-extenders in the U.S. [50], or because Medicaid fee levels for office visits tend to be so much lower than those from other insurers [51]. If so, it is not clear that Medicaid HMOs can fix these underlying problems.

Our findings raise important considerations for state policymakers regarding their growing preference for Medicaid HMOs within Medicaid. There is some evidence that enrollment in Medicaid managed care (including Medicaid HMOs) results in increased healthcare costs and spending for Medicaid recipients and for state Medicaid programs [52, 53]. Additionally, in most states Medicaid HMOs have been documented as having higher overhead than FFS Medicaid, so it is less expensive to administer FFS Medicaid [54].

One of the arguments for Medicaid HMOs has been that the private sector can improve access to care and the quality of care provided to recipients. In line with previous work, as noted earlier, we found that Medicaid recipients in the HMOs were indeed more likely to report having a usual source of care (Table 1), a key indicator of better access to care. Yet, their likelihood of receiving some preventive services was no better than among recipients in FFS Medicaid. Compared to their FFS counterparts, HMO enrollees, surprisingly, had similar rates of flu vaccinations and were less likely to report receiving an annual cholesterol check, or a routine check-up by a physician (Table 1). Thus, at least in terms of these preventive services, there is scant evidence from out data that Medicaid HMOs are providing higher quality care.

Still, states may favor adopting Medicaid HMOs for other reasons. Capitation makes Medicaid spending more predictable. This is critical for policymakers and administrators who often operate within uncompromising political environments and work within strict budgetary constraints. Additionally, contracting with the plans reduces the state’s own administrative burden of Medicaid, even if quality gains are not exceptional or sufficiently realized through such arrangements.

Our paper has shown that potentially preventable ED use is no different under these two models

for Medicaid. Our findings provide an added perspective for assessing and comparing care quality between Medicaid HMOs and Medicaid FFS. However, we believe that more research is needed to more thoroughly evaluate the advantages and disadvantages of Medicaid HMOs, so that policymakers can make more informed decisions regarding the best model to adopt on behalf of vulnerable Medicaid recipients. Future work should focus on comparing additional measures of (1) care quality outcomes, (2) health of Medicaid recipients, (3) risk of mortality, (4) satisfaction with care and coverage, and (5) long term costs associated with each model. In addition to stratifying the analysis on the basis of dual-eligibility status, future work can analyze other special needs and high healthcare utilizing populations. Also, since we were only interested in whether individuals had any preventable ED visit in a year, future work can use alternative modeling strategies to explore the association of Medicaid managed care enrollment and the number of such visits in a year.

### Limitations

Several limitations of our study should be acknowledged. First, since we analyzed secondary data, we are limited in our ability to make causal inferences. However, we tried to approach the question using multiple analytical methods (survey-weighted logistic regression and propensity score weighted logistic regression) to enable us to approach causal inferences. Second, our use of CCCs to identify potentially preventable ED visits likely flags more ED visits as potentially preventable than there are in reality because each CCC includes several ICD-9 codes, some of which may not be considered preventable according to AHRQ guidelines. Further, in our sensitivity analysis we relied on 3-digit ICD-9 codes to identify visits to the ED since the MEPS public-use files do not contain the full 5-digit codes. This may have caused some measurement error because more visits were identified as potentially preventable than in reality. However, recent studies have used a similar operationalization [55–57]. Third, due to the unavailability of 5-digit ICD-9 codes in our data, we were unable to consider other ways of classifying potentially preventable ED visits. For example, we were unable to identify emergent vs. non-emergent ED visits before categorizing potentially preventable and non-preventable ED visits. Fourth, to protect each respondent’s confidentiality, information on their location was absent from the publicly available MEPS files, so we were unable to control for the Medicaid recipient’s state of residence. This is important since Medicaid plans are state specific and unique to their own context. Different states have different rules about Medicaid managed care enrollment, as such pooling the rates across states and modeling national level data, could have masked differences between FFS and managed care recipients in potentially preventable ED. Fifth, due to lack of this information, we also were unable to control for overall managed care penetration in the recipient’s state, as managed care penetration rate has also been shown to be correlated with managed care outcomes. Sixth, we kept the last year of our analysis as 2015, because, currently, the MEPS HC public files do not report either the CCCs or ICD-9 codes associated with ED visits for subsequent years. Seventh, self-reported Medicaid HMO enrollment may be overstated, but likely not in any way that would bias our findings [58]. Finally, our design did not allow for measurement of quality of primary care which is potentially a confounder and likely a mediator for the association between HMO and preventable ED use. We believe that future work should consider a framework for better understanding these links.

## Conclusions

In summary, this study examined the prevalence of potentially preventable ED use within the Medicaid population, and whether Medicaid HMOs have any impact on their occurrence. Separate analyses were conducted for dually eligible recipients (individuals with both Medicaid and Medicare) and non-duals. Using the MEPS data from 2003–2015, we found that Medicaid HMOs were not associated with either an increase or a decrease in the occurrence of potentially preventable ED use, when compared with conventional FFS Medicaid.

## Supporting information

S1 Table Characteristics of the Medicaid population with Preventable Emergency Department (ED) visit ages 18–64 by dual-eligibility status^a^. Results are based on aggregated data from the Medical Expenditures Panel Survey^b^.
(DOCX)

S2 Table Logistic regressions for the prevalence of preventable emergency department visit in the Medicaid non-dual^a^ Population, Ages 18–64 (Unweighted N = 6,585). Results are based on aggregated data from the Medical Expenditures Panel Survey^b^.
(DOCX)

S3 Table Logistic regressions for the prevalence of preventable emergency department visit in the Medicaid duala population, Ages 18–64 (N = 1,059). Results are based on aggregated data from the Medical Expenditures Panel Surveyb.
(DOCX)

S4 Table Logistic regressions for the prevalence of preventable emergency department visit in the Medicaid non-duala population using International Classification of Diseases, Ninth Revision (ICD-9 codes), Ages 18–64 (N = 4,579). Results are based on aggregated data from the Medical Expenditures Panel Surveyb.
(DOCX)

S5 Table Logistic regressions for the prevalence of preventable emergency department visit in the Medicaid duala population using International Classification of Diseases, Ninth Revision (ICD-9 codes), Ages 18–64 (N = 726). Results are based on aggregated data from the Medical Expenditures Panel Surveyb.
(DOCX)

S6 Table Logistic regressions for the prevalence of preventable emergency department visit in the Medicaid non-duala population using Clinical Classification Categories (CCCs) codes till the year 2012, Ages 18–64 (N = 4,579). Results are based on aggregated data from the Medical Expenditures Panel Surveyb.
(DOCX)

S7 Table Logistic regressions for the prevalence of preventable emergency department visit in the Medicaid duala population using Clinical Classification Categories (CCCs) codes till the year 2012, Ages 18–64 (N = 726). Results are based on aggregated data from the Medical Expenditures Panel Surveyb.
(DOCX)

S1 Appendix (DOCX)
