# Supplementary material for: Medicaid managed care and preventable emergency department visits in the United States
Source: PLoS One. 2020 Oct 29;15(10):e0240603. doi: 10.1371/journal.pone.0240603 (PMC7595391; doi:10.1371/journal.pone.0240603)
Supplement: S1 Table — Results are based on aggregated data from the Medical Expenditures Panel Surveyb. (DOCX) [file pone.0240603.s001.docx]

S1 Table: Characteristics of the Medicaid population with Preventable Emergency Department (ED) visit ages 18-64 by dual-eligibility status^a^. Results are based on aggregated data from the Medical Expenditures Panel Survey^b^.

|  |  | |  | |  | |  |
| --- | --- | --- | --- | --- | --- | --- | --- |
|  | Non-duals | | Duals | | *p*-value^c^ | |  |
|  | Unweighted  n =18406 | | Unweighted  n = 2361 | |  | |  |
| Variable | % | | % | |  | |  |
|  | |  | |  | |  | |
| **Key Variables** | |  | |  | |  | |
| Preventable ED Visit | | 17.9 | | 25.3 | | <0.0001 | |
| Medicaid HMO | | 49.7 | | 31.2 | | <0.0001 | |
|  | |  | |  | |  | |
| **Demographics** | |  | |  | |  | |
| **Age** | |  | |  | | <0.0001 | |
| 18 to 35 | | 56.1 | | 11.2 | |  | |
| 36 to 55 | | 35.0 | | 57.3 | |  | |
| 56 and above | | 8.9 | | 31.5 | |  | |
| **Region** | |  | |  | | 0.0011 | |
| Northeast | | 22.4 | | 19.9 | |  | |
| Midwest | | 25.2 | | 22.9 | |  | |
| South | | 29.8 | | 38.5 | |  | |
| West | | 22.7 | | 18.7 | |  | |
| **Male** | | 27.2 | | 39.4 | | <0.0001 | |
| **Education** | |  | |  | | 0.0007 | |
| High School or Less | | 32.3 | | 27.4 | |  | |
| Some College | | 46.9 | | 45.3 | |  | |
| College or More | | 20.8 | | 27.3 | |  | |
| **Income** | |  | |  | | 0.2358 | |
| Poor | | 53.6 | | 52.4 | |  | |
| Near Poor | | 9.7 | | 12.3 | |  | |
| Low Income | | 19.4 | | 18.9 | |  | |
| Middle or High Income | | 17.4 | | 16.4 | |  | |
|  | |  | |  | |  | |
| **Health and Functional Status** | |  | |  | |  | |
| **Self Reported Health** | |  | |  | | <0.0001 | |
| Excellent Health | | 13.3 | | 3.7 | |  | |
| Very Good Health | | 21.5 | | 9.6 | |  | |
| Good Health | | 29.7 | | 21.9 | |  | |
| Fair or Poor Health | | 35.5 | | 64.9 | |  | |
| **Self Reported Mental Health** | |  | |  | | <0.0001 | |
| Excellent Mental Health | | 26.8 | | 10.3 | |  | |
| Very Good Mental Health | | 21.3 | | 16.8 | |  | |
| Good Mental Health | | 30.2 | | 31.4 | |  | |
| Fair or Poor Mental Health | | 21.7 | | 41.4 | |  | |
| **Received help or supervision for instrumental activities of daily living** | | 8.3 | | 27.7 | | <0.0001 | |
| **Received help or supervision for activities of daily living** | | 4.2 | | 13.3 | | <0.0001 | |
| **BMI** | |  | |  | | 0.0002 | |
| Underweight | | 2.8 | | 1.6 | |  | |
| Normal | | 28.8 | | 21.3 | |  | |
| Overweight | | 26.1 | | 28.2 | |  | |
| Obese | | 42.3 | | 48.8 | |  | |
| **Currently Smoke** | | 40.4 | | 44.7 | | 0.0550 | |
| **Access to usual source of care** | | 76.6 | | 92.5 | | <0.0001 | |
|  | |  | |  | |  | |
| **Preventive Care Services Utilization** | |  | |  | |  | |
| Cholesterol Check (more than a year or never) | | 43.7 | | 16.5 | | <0.0001 | |
| Flu Shot (more than a year or never) | | 68.4 | | 44.6 | | <0.0001 | |
| Routine Check (more than a year or never) | | 31.2 | | 14.4 | | <0.0001 | |
| Advised by Doctor to Restrict Fatty Food | | 33.7 | | 53.3 | | <0.0001 | |
| Advised by Doctor to Exercise More | | 43.5 | | 58.8 | | <0.0001 | |
|  | |  | |  | |  | |
| **Attitudes towards health insurance and risk** | |  | |  | |  | |
| **Agree With Following Statements** | |  | |  | |  | |
| Do not need health insurance | | 15.1 | | 7.6 | | <0.0001 | |
| Health insurance is not worth the money it costs | | 41.1 | | 33.4 | | 0.0001 | |
| More likely to take risks | | 40.7 | | 37.7 | | 0.1550 | |
| Can overcome illness without help from a medically trained person | | 29.3 | | 18.2 | | <0.0001 | |
|  | |  | |  | |  | |
| **Clinical Conditions** | |  | |  | |  | |
| Diabetes | | 12.1 | | 29.9 | | <0.0001 | |
| Asthma | | 21.6 | | 29.2 | | 0.0001 | |
| High Blood Pressure | | 32.4 | | 63.9 | | <0.0001 | |
| Coronary Heart Disease | | 5.3 | | 14.4 | | <0.0001 | |
| Angina | | 3.5 | | 9.5 | | <0.0001 | |
| Myocardial Infarction | | 4.9 | | 12.5 | | <0.0001 | |
| Any other heart disease/condition | | 11.4 | | 27.1 | | <0.0001 | |
| Stroke | | 5.2 | | 14.0 | | <0.0001 | |
| Emphysema | | 3.7 | | 13.0 | | <0.0001 | |

a. Dual Eligibility Status: Non-Duals are Medicaid recipients whose only health insurance is Medicaid. Duals are Medicaid

recipients who are also insured through Medicare.

b. Data source: Public use data files from the Medical Expenditure Panel Survey (MEPS) for 2003 through 2015.

c. p-values from Survey Design Based F-test
