# Supplementary material for: Medicaid managed care and preventable emergency department visits in the United States
Source: PLoS One. 2020 Oct 29;15(10):e0240603. doi: 10.1371/journal.pone.0240603 (PMC7595391; doi:10.1371/journal.pone.0240603)
Supplement: S6 Table — Results are based on aggregated data from the Medical Expenditures Panel Surveyb. (DOCX) [file pone.0240603.s006.docx]

S6 Table: Logistic Regressions for the Prevalence of Preventable Emergency Department Visit in the Medicaid Non-Duala Population using Clinical Classification Categories (CCCs) codes till the year 2012, Ages 18-64 (N=4,579). Results are based on aggregated data from the Medical Expenditures Panel Surveyb .

|  | (Model 1) | (Model 2) |
| --- | --- | --- |
| VARIABLES | Survey Logistic Regression^c^ | PSW Logistic Regression^d^ |
|  | Odds Ratio (95% CI^e^) | Odds Ratio (95% CI) |
|  |  |  |
| **Medicaid HMO^f^ (**Ref^g^: Medicaid non-HMO**)** | 0.942 (0.770 - 1.154) | 0.952 (0.776 - 1.167) |
|  |  |  |
| **Demographic Characteristics** |  |  |
| **Age (**Ref: less than 35**)** |  |  |
| 36 to 55 | 0.981 (0.731 - 1.317) | 0.964 (0.712 - 1.304) |
| 56 and above | 0.982 (0.673 - 1.433) | 0.952 (0.645 - 1.404) |
| **Region (**Ref: Northeast**)** |  |  |
| Midwest | 0.898 (0.629 - 1.281) | 0.960 (0.667 - 1.381) |
| South | 1.238 (0.900 - 1.702) | 1.269 (0.909 - 1.771) |
| West | 1.102 (0.777 - 1.563) | 1.122 (0.783 - 1.609) |
| **Male (**Ref: Female**)** | 0.687*** (0.540 - 0.875) | 0.693*** (0.539 - 0.891) |
| **Education (**Ref: High School or Less**)** |  |  |
| Some college | 0.935 (0.750 - 1.166) | 0.932 (0.743 - 1.169) |
| College or more | 1.001 (0.715 - 1.403) | 1.016 (0.724 - 1.426) |
| **Income (**Ref: Poor**)** |  |  |
| Near Poor | 1.060 (0.754 - 1.491) | 1.057 (0.742 - 1.506) |
| Low income | 1.092 (0.838 - 1.422) | 1.115 (0.855 - 1.453) |
| Middle or High Income | 1.298 (0.949 - 1.775) | 1.322* (0.958 - 1.823) |
| **Health and Functional Status Characteristics** |  |  |
| **Self-Reported Health (**Ref: Excellent**)** |  |  |
| Very Good | 1.168 (0.749 - 1.820) | 1.239 (0.798 - 1.922) |
| Good | 1.079 (0.692 - 1.685) | 1.161 (0.739 - 1.825) |
| Fair or Poor | 1.335 (0.864 - 2.063) | 1.446* (0.933 - 2.239) |
| **Self-Reported Mental Health (**Ref: Excellent**)** |  |  |
| Very Good | 0.991 (0.711 - 1.383) | 1.069 (0.762 - 1.500) |
| Good | 1.000 (0.736 - 1.358) | 1.038 (0.752 - 1.432) |
| Fair or Poor | 0.896 (0.646 - 1.241) | 0.947 (0.680 - 1.319) |
| **Received help or supervision for instrumental activities of daily living (**Ref: Otherwise**)** | 0.816 (0.558 - 1.194) | 0.776 (0.527 - 1.143) |
| **Received help or supervision for activities of daily living (**Ref: Otherwise**)** | 1.083 (0.670 - 1.750) | 1.131 (0.691 - 1.852) |
| **BMI (**Ref: Underweight**)** |  |  |
| Normal weight | 0.874 (0.482 - 1.587) | 0.725 (0.385 - 1.364) |
| Overweight | 0.694 (0.378 - 1.275) | 0.582* (0.307 - 1.105) |
| Obese | 0.624 (0.343 - 1.137) | 0.501** (0.270 - 0.930) |
| **Current Smoker (**Ref: Current Non-smoker**)** | 1.002 (0.795 - 1.263) | 0.959 (0.765 - 1.203) |
| **Access to usual source of care (**Ref: No Access**)** | 1.031 (0.778 - 1.365) | 1.009 (0.758 - 1.342) |
| **Preventive Care Services Utilization** |  |  |
| Cholesterol Check^h^ (more than a year or never) | 0.928 (0.711 - 1.211) | 0.908 (0.690 - 1.194) |
| Flu Shot^h^ (more than a year or never) | 0.944 (0.743 - 1.200) | 0.933 (0.731 - 1.191) |
| Routine Check^h^ (more than a year or never) | 0.973 (0.747 - 1.267) | 1.022 (0.780 - 1.338) |
| Advised by Doctor to Restrict Fatty Food (Ref: Doctor did not advice) | 0.925 (0.688 - 1.244) | 0.901 (0.671 - 1.209) |
| Advised by Doctor to Exercise More (Ref: Doctor did not advice) | 1.104 (0.840 - 1.453) | 1.104 (0.836 - 1.458) |
| **Attitudes towards health insurance and risk** |  |  |
| **Agree with following statements^i^** | 1.233 (0.891 - 1.706) | 1.222 (0.877 - 1.702) |
| Do not need health insurance | 0.979 (0.790 - 1.212) | 0.961 (0.771 - 1.197) |
| Health Insurance is not worth the money it costs | 1.083 (0.867 - 1.354) | 1.115 (0.892 - 1.393) |
| More likely to take risks | 0.678*** (0.519 - 0.885) | 0.691*** (0.528 - 0.905) |
| Can Overcome Illness without help from a medically trained person |  |  |
| **Clinical Conditions^j^** | 2.163*** (1.536 - 3.045) | 2.335*** (1.634 - 3.338) |
| Diabetes | 2.323*** (1.863 - 2.896) | 2.271*** (1.814 - 2.842) |
| Asthma | 1.565*** (1.191 - 2.056) | 1.625*** (1.219 - 2.167) |
| High Blood Pressure | 1.253 (0.800 - 1.962) | 1.286 (0.822 - 2.013) |
| Coronary Heart Disease | 1.372 (0.771 - 2.444) | 1.276 (0.718 - 2.268) |
| Angina | 1.161 (0.711 - 1.897) | 1.167 (0.711 - 1.916) |
| Myocardial Infarction | 1.209 (0.863 - 1.694) | 1.147 (0.820 - 1.606) |
| Any other heart disease/condition | 0.792 (0.537 - 1.168) | 0.732 (0.501 - 1.068) |
| Stroke | 1.764** (1.074 - 2.895) | 1.904** (1.118 - 3.242) |
| Emphysema | 0.170*** (0.0795 - 0.365) | 0.185*** (0.0836 - 0.411) |
| Constant | 0.942 (0.770 - 1.154) | 0.952 (0.776 - 1.167) |
|  |  |  |

Notes: ***significant at the 1% level; **significant at the 5% level; and *significant at the 10% level

a. Duals are Medicaid recipients who are also insured through Medicare.

b. Data source: Public use data files from the Medical Expenditure Panel Survey (MEPS) for 2003 through 2012.

c. Logistic regression using survey weights

d. Logistic regression using survey weights combined with propensity score weights

e. CI = Confidence Interval

f. HMO = Health Maintenance Organization

g. Reference category

h. Reference = within last year

i. For all attitudes towards health insurance and risk statements: Reference = Disagree with the statement

j. For all clinical conditions: Reference = Condition not present
