# Supplementary material for: Medicaid managed care and preventable emergency department visits in the United States
Source: PLoS One. 2020 Oct 29;15(10):e0240603. doi: 10.1371/journal.pone.0240603 (PMC7595391; doi:10.1371/journal.pone.0240603)
Supplement: S1 Appendix — (DOCX) [file pone.0240603.s008.docx]

**Data Files Used in the Study**Dataset: Medical Expenditure Panel Survey (MEPS)

**A: Household Component (HC) Full-Year files: Full Year Consolidated Data Files (2003-2015)**
MEPS HC-097: 2005 Full Year Consolidated Data File available at <https://www.meps.ahrq.gov/mepsweb/data_stats/download_data_files_detail.jsp?cboPufNumber=HC-097>

MEPS HC-105: 2006 Full Year Consolidated Data File available at <https://www.meps.ahrq.gov/mepsweb/data_stats/download_data_files_detail.jsp?cboPufNumber=HC-105>

MEPS HC-113: 2007 Full Year Consolidated Data File available at <https://www.meps.ahrq.gov/mepsweb/data_stats/download_data_files_detail.jsp?cboPufNumber=HC-113>

MEPS HC-121: 2008 Full Year Consolidated Data File available at <https://www.meps.ahrq.gov/mepsweb/data_stats/download_data_files_detail.jsp?cboPufNumber=HC-121>

MEPS HC-129: 2009 Full Year Consolidated Data File available at <https://www.meps.ahrq.gov/mepsweb/data_stats/download_data_files_detail.jsp?cboPufNumber=HC-129>

MEPS HC-138: 2010 Full Year Consolidated Data File available at <https://www.meps.ahrq.gov/mepsweb/data_stats/download_data_files_detail.jsp?cboPufNumber=HC-138>

MEPS HC-147: 2011 Full Year Consolidated Data File available at <https://www.meps.ahrq.gov/mepsweb/data_stats/download_data_files_detail.jsp?cboPufNumber=HC-147>

MEPS HC-155: 2012 Full Year Consolidated Data File available at <https://www.meps.ahrq.gov/mepsweb/data_stats/download_data_files_detail.jsp?cboPufNumber=HC-155>

MEPS HC-163: 2013 Full Year Consolidated Data File available at <https://www.meps.ahrq.gov/mepsweb/data_stats/download_data_files_detail.jsp?cboPufNumber=HC-163>

MEPS HC-171: 2014 Full Year Consolidated Data File available at <https://www.meps.ahrq.gov/mepsweb/data_stats/download_data_files_detail.jsp?cboPufNumber=HC-171>

MEPS HC-181: 2015 Full Year Consolidated Data File available at <https://www.meps.ahrq.gov/mepsweb/data_stats/download_data_files_detail.jsp?cboPufNumber=HC-181>


**B: Household Component Event files: Emergency Room Visits files (2003-2015)**
MEPS HC-077E: 2003 Emergency Room Visit file available at <https://www.meps.ahrq.gov/mepsweb/data_stats/download_data_files_detail.jsp?cboPufNumber=HC-077E>

MEPS HC-085E: 2004 Emergency Room Visit file available at <https://www.meps.ahrq.gov/mepsweb/data_stats/download_data_files_detail.jsp?cboPufNumber=HC-085E>

MEPS HC-094E: 2005 Emergency Room Visit file available at <https://www.meps.ahrq.gov/mepsweb/data_stats/download_data_files_detail.jsp?cboPufNumber=HC-094E>

MEPS HC-102E: 2006 Emergency Room Visit file available at <https://www.meps.ahrq.gov/mepsweb/data_stats/download_data_files_detail.jsp?cboPufNumber=HC-102E>

MEPS HC-110E: 2007 Emergency Room Visit file available at <https://www.meps.ahrq.gov/mepsweb/data_stats/download_data_files_detail.jsp?cboPufNumber=HC-110E>

MEPS HC-118E: 2008 Emergency Room Visit file available at <https://www.meps.ahrq.gov/mepsweb/data_stats/download_data_files_detail.jsp?cboPufNumber=HC-118E>

MEPS HC-126E: 2009 Emergency Room Visit file available at <https://www.meps.ahrq.gov/mepsweb/data_stats/download_data_files_detail.jsp?cboPufNumber=HC-126E>

MEPS HC-135E: 2010 Emergency Room Visit file available at <https://www.meps.ahrq.gov/mepsweb/data_stats/download_data_files_detail.jsp?cboPufNumber=HC-135E>

MEPS HC-144E: 2011 Emergency Room Visit file available at <https://www.meps.ahrq.gov/mepsweb/data_stats/download_data_files_detail.jsp?cboPufNumber=HC-144E>

MEPS HC-152E: 2012 Emergency Room Visit file available at <https://www.meps.ahrq.gov/mepsweb/data_stats/download_data_files_detail.jsp?cboPufNumber=HC-152E>

MEPS HC-160E: 2013 Emergency Room Visit file available at <https://www.meps.ahrq.gov/mepsweb/data_stats/download_data_files_detail.jsp?cboPufNumber=HC-160E>

MEPS HC-168E: 2014 Emergency Room Visit file available at <https://www.meps.ahrq.gov/mepsweb/data_stats/download_data_files_detail.jsp?cboPufNumber=HC-168E>

MEPS HC-178E: 2015 Emergency Room Visit file available at <https://www.meps.ahrq.gov/mepsweb/data_stats/download_data_files_detail.jsp?cboPufNumber=HC-178E>"
